# Overexpression of Kinesin Family Member 20A Correlates with Disease Progression and Poor Prognosis in Human Nasopharyngeal Cancer: A Retrospective Analysis of 105 Patients

**DOI:** 10.1371/journal.pone.0169280

**Published:** 2017-01-12

**Authors:** Sai-Lan Liu, Huan-Xin Lin, Fang Qiu, Wei-Jing Zhang, Chun-Hao Niu, Wen Wen, Xiao-Qing Sun, Li-Ping Ye, Xian-Qiu Wu, Chu-Yong Lin, Li-Bing Song, Ling Guo

**Affiliations:** 1 Sun Yat-sen University Cancer Center, State Key Laboratory of Oncology in South China, Collaborative Innovation Center for Cancer Medicine, Guangzhou, Guangdong Province, People's Republic of China; 2 Department of Nasopharyngeal Carcinoma, Sun Yat-sen University Cancer Center, Guangzhou, Guangdong Province, People's Republic of China; 3 Department of Radiotherapy, Sun Yat-sen University Cancer Center, Guangzhou, Guangdong Province, People's Republic of China; 4 Department of Gynaecological Oncology, Sun Yat-sen University Cancer Center, Guangzhou, Guangdong Province, People's Republic of China; Taipei Medical University, TAIWAN

## Abstract

**Background:**

Numerous studies have shown Kinesin family member 20A (KIF20A) may play a critical role in the development and progression of cancer. However, the clinical value of KIF20A in nasopharyngeal carcinoma (NPC) is unknown. Here, we investigated the expression pattern of KIF20A in NPC and its correlation with clinicopathological features of patients.

**Methods:**

Real-time PCR and Western blotting were used to quantify KIF20A expression in NPC cell lines and clinical specimens compared with normal controls. KIF20A protein expression was also examined in archived paraffin embedded tumor samples from 105 patients with pathologically confirmed NPC by immunohistochemistry (IHC). Statistical analyses were applied to assess the associations between KIF20A expression and the clinicopathological features and survival outcomes. Effects on migration and invasion were assessed by wound healing and transwell invasion assays after KIF20A silencing.

**Results:**

KIF20A was significantly overexpressed at both the mRNA and protein levels in NPC cell lines and human tumor tissues. 45/105 (42.9%) of NPC specimens expressed high levels of KIF20A among the KIF20A detectable cases. Statistical analysis revealed that high KIF20A expression was significantly associated with gender (*P* = 0.046), clinical stage (*P*<0.001), T category (*P* = 0.022), N category (*P*<0.001), distant metastasis (*P* = 0.001) and vital status (*P* = 0.001). Moreover, Higher KIF20A expression patients had shorter overall survival (OS) and progression-free survival (PFS) (P = 0.001 and P = 0.001; log-rank test). In multivariate analysis, KIF20A was an independent prognostic factor for OS and PFS in the entire cohort (*P* = 0.033, *P* = 0.008). Knock down of KIF20A expression significantly suppressed NPC cell’s migration and invasion.

**Conclusions:**

KIF20A is overexpressed and may serve as an independent prognostic biomarker in NPC. Targeting KIF20A reduces migration and invasion of NPC cells.

## Background

Nasopharyngeal carcinoma (NPC) differs from malignant tumors arising from other mucosal sites in the head and neck in terms of its unique epidemiology, pathological types and therapeutic management[1,2]. NPC has a unique ethnic and geographical distribution, with an extremely high incidence in Guangdong province of Southern China, where environmental factors, genetic predisposition and Epstein-Barr virus (EBV) infection have been found to play important roles in the pathogenesis of this disease[3,4].

Concurrent chemoradiotherapy (CCRT) with cisplatin-based regimens is the standard treatment for NPC and the widespread adoption of intensity-modulated radiotherapy (IMRT) has resulted in excellent locoregional control rates [5–14]. Although advances have been made the clinical treatment of NPC, the outcome for patients with locoregionally advanced disease remains inadequate[15]. Local recurrence and metastasis remain the most common causes of mortality in advanced stage disease [16–18].Therefore, novel biomarkers associated with diagnosis and disease progression urgently need to be discovered in order to identify high-risk patients who could benefit from more aggressive clinical strategies.

Dysregulation of the cell cycle can promote cancer cell growth and proliferation. Cell cycle alterations have been attributed to a variety of molecules including the kinesins, which are important for mitosis. Sixteen mitotic kinesins have been identified to play crucial roles in the development and progression of various types of cancer[19]. Kinesin family member 20A (KIF20A, also known as RAB6KIFL) belongs to kinesin superfamily-6 and contains a conserved motor domain. KIF20A was first identified to localize to the Golgi apparatus and participate in organelle dynamics by interacting with the GTP-bound form of Rab6[20]. KIF20A binds to microtubules to generate mechanical force by coupling with adenosine triphosphate hydrolysis[21].

The testes and thymus are the only tissues that normally express KIF20A[22]. Numerous studies have shown KIF20A may play a critical role in the development and progression of cancer. KIF20A was found to be consistently overexpressed in pancreatic cancer in several different high-throughput expression profiling analyses, and a previous study demonstrated that targeting KIF20A reduces the proliferation, migration and invasion of pancreatic cancer cells[23,24]. KIF20A has also been reported to be overexpressed in other types of cancer, including bladder cancer, gastric cancer, hepatocellular carcinoma, melanoma and breast cancer[25–29]. However, the expression and role of KIF20A in NPC have not yet been examined.

In this study, we report that KIF20A is frequently overexpressed in NPC and is significantly associated with advanced stage disease as well as poorer overall survival (OS) and progression-free survival (PFS). Knock down of KIF20A expression significantly suppressed NPC cell’s migration and invasion.

Taken together, KIF20A may represent a useful biomarker in NPC progression and targeting KIF20A reduces migration and invasion of NPC cells.

## Materials and Methods

### Aim, design and setting of study

We aimed to assess KIF20A expression in NPC cell lines and human tissues and examine the relationship between KIF20A and the clinicopathological features and outcomes of patients with NPC. This study employed both in vitro analysis in conjunction with a retrospective analysis of clinical specimens obtained from 105 patients treated at a single institution in China.

### Microarray data process

Microarray data process and visualization microarray data sets (GEO accession number: GSE12452, GSE13597, GSE53819) from NPC samples, and control samples were retrieved from the GEO database (http://www.ncbi.nlm.nih.gov/geo/). We subsequently performed integrative analyses on the Cancer Genome Atlas (TCGA) data for Head and Neck Squamous Cell Carcinoma (TCGA, Nature 2015).

### Cell lines

The primary normal nasopharyngeal epithelial cell line NP69 which obtained from Dr. George SW Tsao, Cancer Center, Hong Kong University, Hong Kong, was cultured in keratinocyte/serum-free medium (Invitrogen, Grand Island, NY, USA). Human NPC cells CNE1, CNE2, HK1, SUNE1, CNE-2 subclones S18 and S26, and SUNE1 subclones 5-8F and 6-10B, were originated from primary culture of epithelial cells from human NPC bioptic specimen and cultured in RPMI 1640 (Invitrogen) supplemented with 10% fetal bovine serum (HyClone, Logan, UT), 100 μg/μL streptomycin and 100 μg/μL penicillin.

### Tissue specimens and patient information

A total of 105 cases of paraffin-embedded NPC tissue samples had been clinically and histologically diagnosed at the Sun Yat-Sen University Cancer Center between 2006 and 2010. This study was approved by the Institutional Clinical Ethics Review Board of Sun Yat-sen University Cancer Center, and written informed consent was obtained from each patient. Tumor grade and stage were classified according to the seventh edition of the Union for International Cancer Control (UICC) staging system.

Two (1.9%) of the 105 patients had stage I disease; 19 (18.1%), stage II; 52 (49.5%), stage III; and 32 (30.5%), stage IV. 18 patients received radiotherapy and 87 patients received concurrent chemoradiotherapy. The clinicopathological features of the patients are summarized in Table 1. No patients had distant metastasis at diagnosis of NPC. The follow-up time of the cohort ranged from 3 to 94 months, with a median follow-up time of 64 months.

**Table 1:** Association between KIF20A expression and the clinicopathological features of 105 with patients NPC.

Feature	Total	KIF20A		P-value
		Low expression	High expression	
**Age (years)**				
≥45	45 (42.9%)	30 (55.6%)	24 (44.4%)	0.735^†^
<45	60(57.1%)	30(58.8%)	21(41.2%)	
**Gender**				
Male	78 (74.3%)	49 (62.8%)	29 (37.2%)	0.046^†^
Female	27 (25.7%)	11 (40.7%)	16 (62.8%)	
**Histologic classification**				
U	102 (97.1%)	58 (56.9%)	44 (43.1%)	0.888*
D	3 (2.9%)	2 (66.7%)	1 (33.3%)	
**T classification**				
1	7 (6.7%)	6 (85.7%)	1 (14.3%)	0.022^†^
2	24 (22.9%)	17 (70.8%)	7 (29.2%)	
3	52 (49.5%)	29 (55.8%)	23 (44.2%)	
4	22 (21.0%)	8(36.4%)	14 (63.6%)	
**N classification**				
0	17 (16.2%)	16 (94.1%)	1 (2.2%)	<0.001^&^
1	50 (47.6%)	36 (72%)	14(28%)	
2	27(25.7%)	8(29.6%)	19(70.4%)	
3	11(10.5%)	0 (0%)	11(100%)	
**Clinical stage**				
I	2(1.9%)	2 (100.0%)	0 (0.0%)	<0.001^&^
II	19 (18.1%)	17 (89.5%)	2 (10.5%)	
III	52 (49.5%)	33 (63.5%)	19(36.5%)	
IV	32 (30.5%)	8 (25.0%)	24 (75.0%)	
**Metastasis**				
No	89 (84.8%)	57(64.0%)	32 (36.0%)	0.001^†^
Yes	16 (15.2%)	3(18.8%)	13(81.2%)	
**Vital status**				
Alive	91 (86.7%)	58 (63.7%)	33(36.3%)	0.001*
Dead	14 (13.3%)	2 (14.3%)	12 (85.7%)	
**Treatment method**				
Radiotherapy	18(17.1%)	14(77.8%)	4(22.2%)	0.052^†^
CCRT	87(82.9%)	46(52.9%)	41(47.1%)	

^†^P values, *P values, ^&^P values were calculated with the chi-square test, Continuity Correlation and the Fisher’s exact test, respectively.

Abbreviations: NPC, nasopharyngeal carcinoma; U, undifferentiated non-keratinized carcinoma; D,differentiated non-keratinized carcinoma; CCRT, concurrent chemoradiotherapy.

For real-time PCR (RT-PCR) and Western blot analysis, the freshly frozen NPC samples and noncancerous nasopharyngeal samples were obtained from the patients who underwent nasopharyngeal biopsy before treatment after obtaining informed consent. Three paired tumor samples (T1-3) and the adjacent noncancerous tissue samples (N1-3) were obtained from the same patients, and additional three tumor samples (T4-6) were obtained from three other patients with NPC. All specimens were pathologically-confirmed at the Cancer Center of Sun Yat-sen University.

### Real-time PCR

Total RNA samples from cells lines and freshly frozen tissues were isolated using TRIzol reagent (Invitrogen, Carlsbad, CA, USA) following the manufacturer’s recommendations. The extracted RNA was pretreated with RNase-free DNase and 2.0 μg of total RNA was reverse transcribed to complementary DNA (cDNA) using random hexamers. To amplify KIF20A cDNA, the real-time RT-PCR cycling conditions were an initial denaturation step at 95°C for 10 min, followed by 28 cycles of denaturation at 95°C for 60 s, primer annealing at 58°C for 30 s and extension at 72°C for 30 s; followed by a final extension step at 72°C for 5 min, then the products were stored at 4°C. The primers were designed using Primer Express Software v. 2.0 (Applied Biosystems) and had the following sequences: KIF20A, forward 5-TGCTCTGTCGTCTCTACCTCC-3 and reverse 5-TAACAAGGGCCTAACCCTCA-3; and glyceraldehyde-3-phosphate dehydrogenase (GAPDH), forward 5′-AAGGTCATCCCTGAGCTGAA-3′ and reverse 5-TGACAAAGTGGTCGTTGAGG-3′. KIF20A expression levels were normalized to the geometric mean of GAPDH and calculated using 2−[(Ct of KIF20A)−(Ct of GAPDH)], where Ct represents the threshold cycle for each transcript. Each experiment was performed in triplicate.

### Western blotting

Samples were prepared for immunoblotting as previously described. Cells cultured to 70 to 80% confluence were washed twice with ice-cold phosphate-buffered saline (PBS) and lysed on ice in radio immunoprecipitation assay buffer (RIPA; Cell Signaling Technology, Danvers, MA, USA) with complete protease inhibitor cocktail (Roche Applied Sciences, Mannheim, Germany), then heated for10 min at 98°C. The freshly-frozen tissue samples were ground to powder in liquid nitrogen and lysed using SDS-PAGE sample buffer. Protein concentrations were determined with Bradford assay reagent (Bio-Rad Laboratories, Hercules, CA, USA). Equal amounts of protein (30 μg) were separated by 10.5% SDS polyacrylamide gel electrophoresis, transferred to PVDF membranes (Immobilon P; Millipore, Bedford, MA, USA), blocked using 5% fat-free milk in Tris-buffered saline containing 0.1% Tween-20 (TBS-T) for 1 h at room temperature. Membranes were probed with 1:200-diluted anti-KIF20A rabbit polyclonal antibody (Sigma) overnight at 4°C, and then with horseradish peroxidase-conjugated goat anti-rabbit IgG (1:3000). An anti-α-tubulin mouse monoclonal antibody (1:1000; Santa Cruz Biotechnology, Santa Cruz, CA) was used as a loading control. KIF20A expreesion was detected using enhanced chemiluminescence reagent (Amersham Pharmacia Biotech) following the manufacturer’s instructions.

### Immunohistochemical analysis

KIF20A protein expression was examined in the 105 human NPC tissue specimens using immunohistochemistry. Briefly, 4 μm-thick paraffin-embedded sections were baked at 60°C for 1 h. The sections were deparaffinized with xylenes, rehydrated, placed in EDTA antigen retrieval buffer and microwaved. Endogenous peroxidase activity was inhibited using a solution of 3% hydrogen peroxide in methanol, non-specific binding was blocked in 1% bovine serum albumin, and then the sections were incubated with anti-KIF20A rabbit polyclonal antibody (1:50; Sigma) at 4°C overnight. Normal goat serum was used as a negative control instead of primary antibody. After washing, the sections were incubated with biotinylated anti-rabbit secondary antibody (Abcam) followed by incubation with streptavidin horseradish peroxidase complex (Abcam), then developed in 3-amino-9-ethyl carbazole, counterstained using 10% Mayer’s hematoxylin, dehydrated, and mounted in Crystal Mount (Company).

The degree of immunostaining was scored independently by two observers. The proportion of KIF20A-expressing cells was scored as 1 (<25% positive tumor cells), 2 (25–50%), 3 (50–75%), or 4 (> 75%); and the staining intensity as 0 (no staining), 1 (weak, light yellow), 2 (moderate, yellowish brown), and 3 (strong, brown). The scores for the staining intensity and proportion of positive cells in each section were multiplied (to obtain values of 0, 1, 2, 3, 4, 6, 8, 9, or 12). The cutoff value for KIF20A was selected using receiver operating characteristic (ROC) curve analysis. Tumor samples with scores > 6 were classified as having high expression and samples with scores ≤ 6, low KIF20A expression. The AxioVision Rel.4.6 computerized image analysis system, assisted with the automatic measurement program (Carl Zeiss), was used to analyzed the inconsistencies in IHC stain intensities in tumor and normal tissues.

### Small interfering RNA (siRNA) transfection

Using Lipofectamine RNAiMAX (Invitrogen) according to manufacturer's instructions to perform SiRNA transfections. KIF20A siRNAs were purchased from RiboBio (Guangzhou).The targeting sequences were as follows: si-KIF20A-1: 5’-CTCCGAGATGAAATTTGCA -3’; si-KIF20A-2: 5 -GGTCTGTGGTACGCAAGAA-3’; and si-KIF20A-3: 5’- GTCGTAGTTTCTCCCATGT-3’.

### Transwell invasion assay

SUNE1 and HK1 cells were first transfected with KIF20A siRNA or control siRNA. 3x10^4^ transfected cells were seeded on top of a thick layer of Matrigel in transwell inserts (BD Biosciences) and cultured for another 24 hours. Invasive cells adhered to the lower surface of filter were washed with PBS, fixed with 4% paraformaldehyde and stained with 0.05% crystal violet. The invasive cells were counted under a light microscope (Zeiss).

### Wound healing assay

1x10^6^ transfected Cells were seeded and allowed to reach 70%-80% confluence, then starved for 24 hours. The cell monolayers were then wounded with a sterile plastic tip and cultured in serum-free medium. Cell migration was monitored every 12 hours using microscopy (Nikon).

### Statistical analysis

All statistical analyses were conducted using the SPSS 19.0 statistical software packages. The associations between KIF20A expression and clinicopathological features were examined using the Pearson’s χ^2^ tests or Fisher exact tests. OS was measured from initiation of treatment to death; PFS from initiation of treatment to disease progression or censorship at last follow-up. Survival curves were plotted using the Kaplan-Meier method and compared via the log-rank test. Multivariate analysis was performed using Cox’s proportional hazards model. Two-sided *P*-values < 0.05 were considered significant.

## Results

### KIF20A is overexpressed in NPC cell lines and human NPC tissues

**Fig 1:**
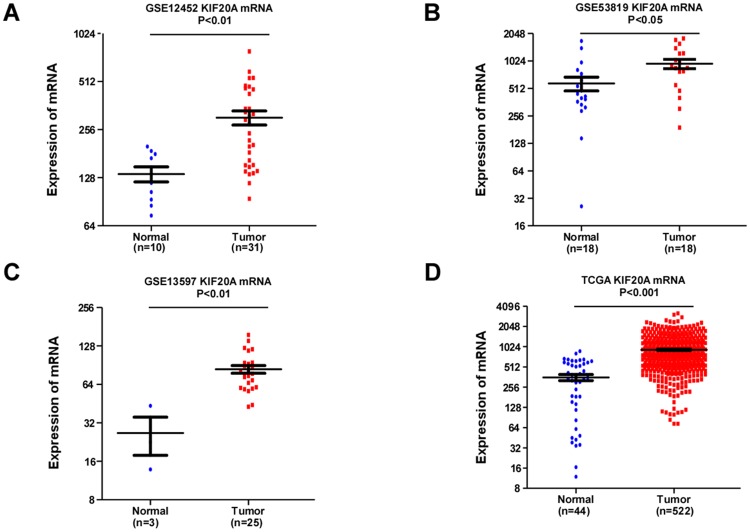
Microarray data reveals KIF20A is upregulated in NPC. (A) Expression of KIF20A in Array Express (GSE12452) NPC and normal tissues (Mann-Whitney test; *P* < 0.01). (B) Expression of KIF20A in Array Express (GSE53819) NPC and normal tissue data (Mann-Whitney test; *P* < 0.05). (C) Expression of KIF20A in Array Express (GSE13597) NPC and normal tissue data (Mann-Whitney test; *P* < 0.01). (D) Expression of KIF20A in TCGA (head and neck) tumor and normal tissue data (Mann-Whitney test; *P* < 0.001).

**Fig 2:**
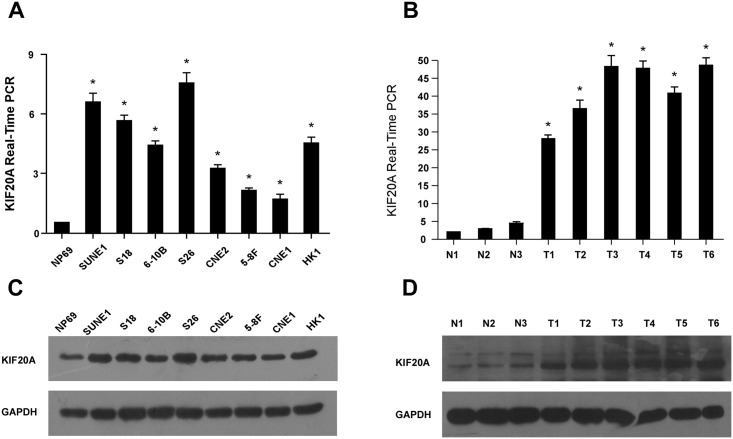
KIF20A is upregulated in NPC cell lines and tissues. (A) Reverse transcription (RT)-PCR and real-time PCR analysis of KIF20A mRNA expression in NP69 immortalized nasopharyngeal epithelial cells and eight cultured NPC cell lines. GAPDH was used as a loading control. * *P* ≤ 0.05. (B) Reverse transcription (RT)-PCR and real-time PCR analysis of KIF20A mRNA expression in three normal nasopharyngeal epithelial biopsies and six NPC tumor samples (three matched samples); GAPDH was used as a loading control. * *P* ≤ 0.05. (C) Western blotting analysis of KIF20A protein expression in NP69 immortalized nasopharyngeal epithelial cells and eight cultured NPC cell lines. GAPDH was used as a loading control. (D) Western blotting analysis of KIF20A protein expression in three normal nasopharyngeal epithelial biopsies and six NPC tumor samples (three matched samples); GAPDH was used as a loading control. Error bars are standard deviation of the mean (SD) calculated from three experiments performed in parallel.

Analysis of publically-available microarray data (GSE12452, GSE13597, GSE53819, and TCGA data for Head and Neck Squamous Cell Carcinoma (HNSCC)) revealed *KIF20A* was upregulated in NPC tumor samples and HNSCC compared with normal tissues (Fig 1A–1D). Similarly, KIF20A mRNA and protein were overexpressed in all eight NPC cell lines tested compared to the normal nasopharyngeal epithelial line NP69 (Fig 2A and 2C).

To confirm whether KIF20A is overexpressed in human NPC, paired tumor samples and the adjacent noncancerous tissues from three patients and tumor samples from three other patients were examined using quantitative real-time PCR and Western blotting. *KIF20A* mRNA and protein expression were significantly upregulated in the six NPC tumor samples compared to the three normal nasopharyngeal tissues (Fig 2B and 2D). Collectively, these results demonstrate KIF20A is upregulated in human NPC.

### KIF20A expression correlates with the clinicopathological features of NPC

Next, we further assessed the association between KIF20A protein expression and the clinicopathological features of NPC using the 105 archived paraffin-embedded human NPC specimens. The stage distribution of the cohort was as follows: two patients had stage I NPC, 19 had stage II NPC, 52 had stage III NPC, and 32 had stage IV NPC.

**Fig 3:**
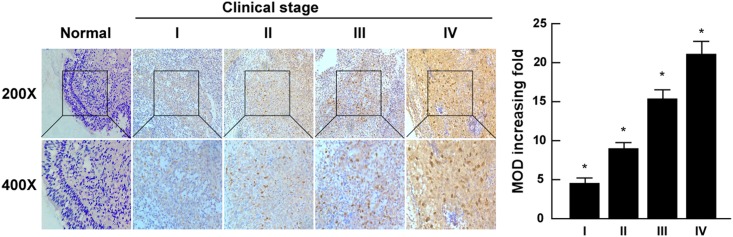
Expression of KIF20A in different clinical stages of NPC. A. Representative images of immunohistochemical staining for KIF20A in normal (control sections) nasopharyngeal tissues and different clinical stages of NPC. B. Average fold change in the mean optical density (MOD) of KIF20A in different clinical stages of NPC compared to normal nasopharyngeal tissues.*P < 0.05.

Immunohistochemistry revealed KIF20A was primarily expressed in the tumor cell nuclei with occasional strong cytoplasmic staining detected. In contrast, KIF20A was barely detected in normal epithelial cells (Fig 3A). Using the cutoff score of ≤ 6, 45/105 (42.9%) of tumor specimens were classified as high KIF20A-expressing and 60/105 (57.1%) as low KIF20A-expressing.

Moreover, KIF20A expression, as assessed using immunohistochemistry, increased with increasing clinical stage in NPC (Fig 3B). Quantitative analysis confirmed that the average mean absorbance value for KIF20A staining was significantly higher in the tumor tissues than the normal nasopharyngeal tissues. In addition, the mean optical density (MOD) values for KIF20A staining significantly increased as tumor stage increased from I to IV (*P* < 0.05, Fig 3B).

The association between KIF20A and the clinicopathological characteristics of NPC were examined using the Pearson’s χ^2^ tests or Fisher exact tests. (Table 1). No significant association was observed between KIF20A expression and age or histologic classification. However, KIF20A expression was significantly associated with gender (*P* = 0.046), clinical stage (*P* < 0.001), T category (*P* = 0.022), N category (*P* < 0.001), distant metastasis (*P* = 0.001) and vital status (*P* = 0.001; Table 1).

### High KIF20A expression is associated with poor survival and prognosis in NPC

**Fig 4:**
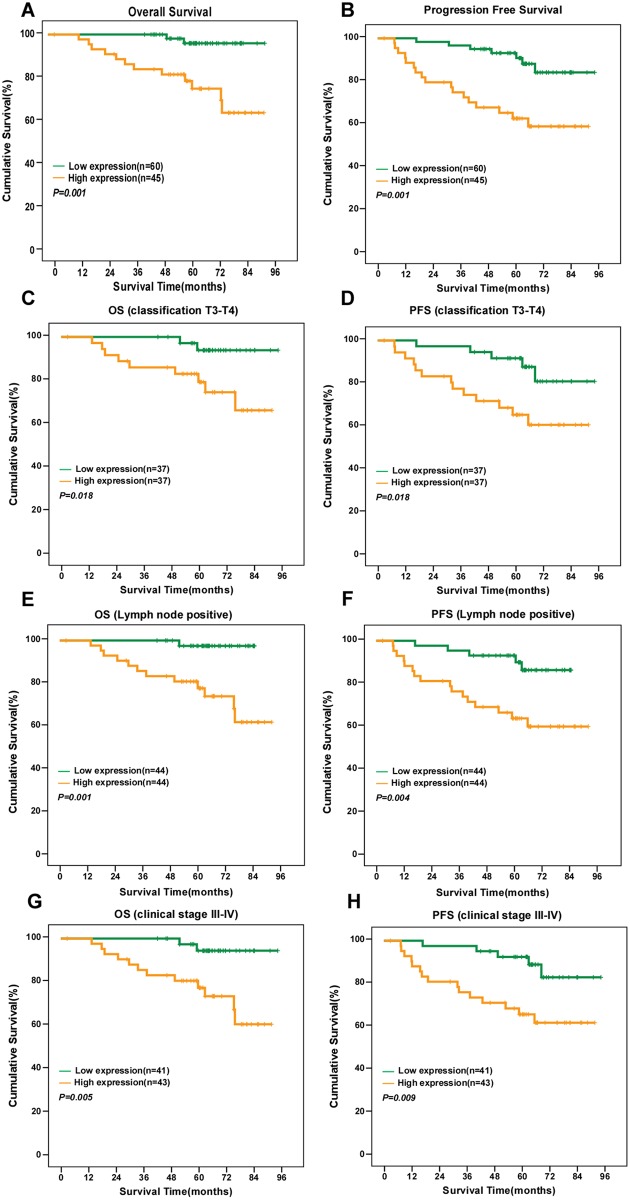
KIF20A protein expression is associated with overall survival and progression-free survival in NPC. (A, B) Kaplan–Meier overall survival (A) and progression-free survival (B) curves for all 105 patients with NPC stratified by high KIF20A expression (*n* = 45) versus low KIF20A expression (*n* = 60). Kaplan–Meier overall survival (C, E, G) and disease-free survival (D, F, H) curves for the subgroups of patients with T3–T4 NPC (C, D), lymph node-positive NPC (E, F) and stage III–IV NPC (G, H) stratified by high and low expression of KIF20A. *P* values were calculated using the log-rank test.

Kaplan-Meier survival analysis revealed high KIF20A protein expression was significantly associated with poor 5-year OS and PFS (*P* = 0.001 and *P* = 0.001; Fig 4A and 4B). Cumulative 5-year OS and PFS for the high KIF20A-expressing group were 78.5% and 62.7%, respectively, and 95.9% and 90.8%, respectively, for the low or no KIF20A-expressing group. To further assess the prognostic value of KIF20A, the patients were stratified into subgroups using stage and T, N, and clinical stage. There was no significant association between KIF20A expression and OS or PFS in the subgroup of patients with subgroup with T1-2 NPC; the subgroup without lymph node metastasis (N0) and early clinical stage disease (stage I–II) (data not shown). However, high KIF20A expression was significantly associated with poor OS and PFS in the subgroups of patients with T3–4 classification (*P* = 0.018 and *P* = 0.018, respectively, Fig 4C and 4D), neck lymph node metastasis (*P* = 0.001 and *P* = 0.004, Fig 4E and 4F), advanced stage (stage III-IV) (*P* = 0.005 and *P* = 0.009, Fig 4G and 4H). Thus, KIF20A seems to be a more valuable prognostic marker in advanced NPC than early-stage disease.

**Fig 5:**
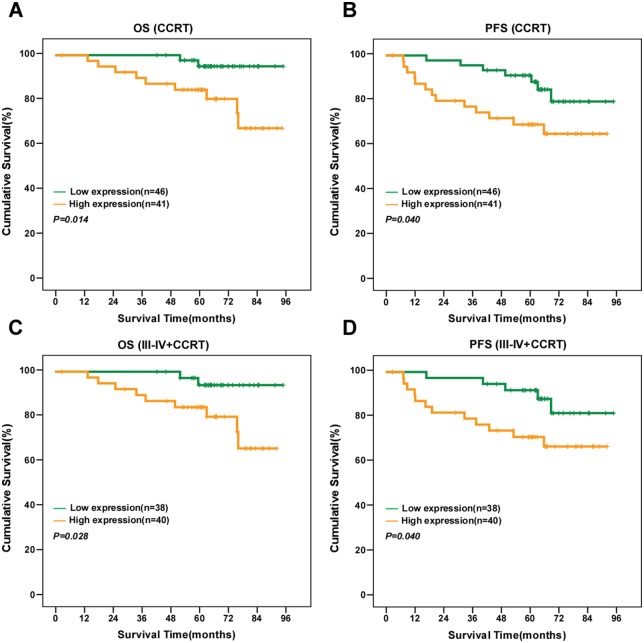
KIF20A protein expression is associated with overall survival and progression-free survival in NPC patients with NPC who received CCRT. Kaplan–Meier overall survival (A,C) and progression-free survival (B,D) curves for the subgroups of patients with NPC who received CCRT (A,B) and the advanced clinical stage III-IV NPC patients who treated with CCRT subgroup(C, D) stratified by high and low expression of KIF20A. *P* values were calculated using the log-rank test.

In addition, patients with high KIF20A expression in the concurrent chemotherapy and radiotherapy(CCRT) subgroups had significantly poorer OS and PFS than the patients with low/no KIF20A expression in the same subgroup (*P* = 0.014 and *P* = 0.040, respectively; Fig 5A and 5B). What’s more, we found that higher KIF20A expression was correlated with a significantly shorter OS and PFS in the advanced clinical stage III-IV NPC patients who treated with CCRT subgroup (*P* = 0.028 and *P* = 0.040, respectively; Fig 5C and 5D).The data demonstrated that the advanced clinical stage III-IV NPC patients who overexpress KIF20A still had a poor survival even after had treated with CCRT.

### KIF20A is an independent prognostic factor for clinical outcome in NPC

In multivariate analysis to adjust for a variety of risk factors, high KIF20A expression remained an independent prognostic factor for poor OS (HR: 6.229; 95% CI: 1.158–33.516; *P* = 0.033) and PFS (HR: 4.129; 95% CI: 1.450–11.757; *P* = 0.008; Table 2) in the whole cohort. Furthermore, multivariate survival analysis was performed in the subgroup of patients with advanced clinical stage III-IV.

**Table 2:** Univariate and Multivariate Cox regression analysis of the association of various clinicopathological features with overall survival and progression-free survival in 105 patients with NPC.

Feature	Univariate		Multivariate	
	Regression coefficient(SE)	P	Hazard ratio(95%CI)	P
**OS**				
Age (y) ≥45 vs <45	0.281 (0.542)	0.604	-	-
Gender M VS F	-0.446 (0.558)	0.640	-	-
T stage TI VS II VS III VS IV	0.168 (0.336)	0.618	-	-
N stage N0 VS I VS II VS III	0.782 (0.312)	0.012	1.264 (0.628–2.544)	0.512
Clinical stage I VS II VS III VS IV	0.860 (0.423)	0.042	1.259 (0.499–3.179)	0.626
RadiotherapyVS CCRT	-0.329 (0.653)	0.0615	-	-
KIF20A expression Low VS High	2.189 (0.764)	0.004	6.229(1.158–33.516)	0.033
**PFS**				
Age (y) ≥45 vs <45	0.074 (0.410)	0.856	-	-
Gender M VS F	-0.291 (0.433)	0.502	-	-
T stage TI VS II VS III VS IV	0.253 (0.264)	0.339	-	-
N stage N0 VS I VS II VS III	0.462 (0.240)	0.054	0.987 (0.555–1.755)	0.964
Clinical stage I VS II VS III VS IV	0.507 (0.305)	0.096	-	-
Radiotherapy VS CCRT	0.010 (0.548)	0.986	-	-
KIF20A expression Low VS High	1.405 (0.450)	0.002	4.129(1.450–11.757)	0.008

Abbreviations: NPC, nasopharyngeal carcinoma; M, male; F, female; CCRT, concurrent chemoradiotherapy; OS, overall survival; PFS, progression free survival.

KIF20A expression (P = 0.010 and P = 0.046, respectively; Table 3) was still recognized as independent prognostic factors for OS and PFS.

**Table 3:** Univariate and Multivariate Cox regression analysis of the association of various clinicopathological features with overall survival and progression-free survival in 84 clinical stage III-IV NPC patients.

Feature	Univariate		Multivariate	
	Regression coefficient (SE)	P	Hazard ratio(95%CI)	P
**OS**				
Age (y) ≥45 vs <45	0.065 (0.541)	0.905	-	-
Gender M VS F	-0.345 (0.559)	0.537	-	-
T stage TI VS II VS III VS IV	-0.4148 (0.383)	0.280	-	-
N stage N0 VS I VS II VS III	0.581 (0.326)	0.074	-	-
Clinical stage III VS IV	0.400 (0.542)	0.461	-	-
Radiotherapy VS CCRT	-1.720 (0.670)	0.010	0.142 (0.037–0.541)	0.004
KIF20A expression Low VS High	1.882 (0.765)	0.014	7.329 (1.622–33.129)	0.010
**PFS**				
Age (y) ≥45 vs <45	-0.115 (0.450)	0.798	-	-
Gender M VS F	0.004 (0.488)	0.993	-	-
T stage TI VS II VS III VS IV	0.224 (0.365)	0.540	-	-
N stage N0 VS I VS II VS III	0.480 (0.272)	0.078	-	-
Clinical stage III VS IV	0.892 (0.451)	0.048	1.726 (0.683–4.365)	0.249
Radiotherapy VS CCRT	-1.018 (0.628)	0.105	-	-
KIF20A expression Low VS High	1.269 (0.517)	0.014	2.961 (1.022–8.580)	0.046

Abbreviations: NPC, nasopharyngeal carcinoma; M, male; F, female; CCRT, concurrent chemoradiotherapy; OS, overall survival; PFS, progression free survival.

Taken together, these results indicate KIF20A could represent a valuable independent prognostic marker of treatment outcome in NPC, especially in the subgroup of patients with advanced clinical stage III-IV.

### Knock down of KIF20A suppressed migration and invasion in NPC cell lines

**Fig 6:**
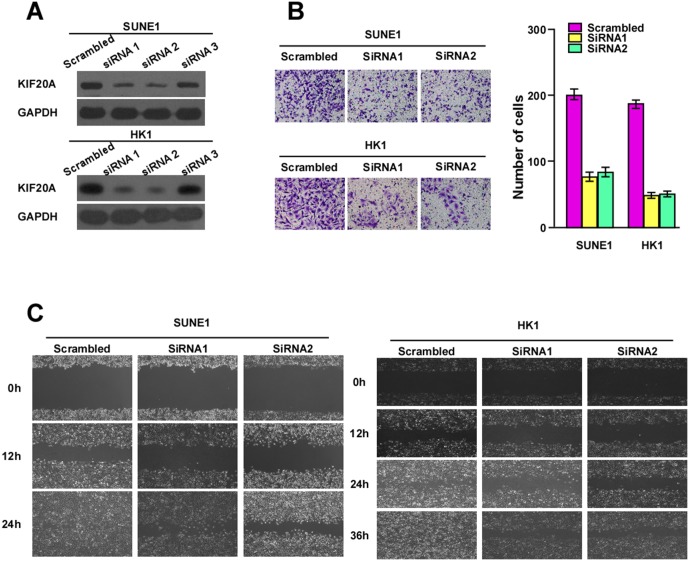
Effects of KIF20A silencing on cancer cell migration and invasion. (A)The knock-down efficiency of endogenous KIF20A expression on protein levels were determined by western blotting analysis. (B) SUNE1 and HK1 cells were transfected with two individual KIF20A siRNAs or a scrambled control followed by transwell invasion assays. (C) Effect on migration by wound healing assays. Data are presented as the mean ± SD, and chance was ruled out with Student’s t test.

As a motor protein, KIF20A has kinetic functions in cells, namely, cell division and motility. We therefore silenced KIF20A in SUNE1 and HK1 cells where it was overexpressed and assessed cell motility. Efficient knock-down of KIF20A expression was confirmed by western blotting (Fig 6A), with more efficient knock-down by siRNA 1 and siRNA 2. As shown in Fig 6B, knock-down of KIF20A expression strongly reduced numbers of invasive cells. As expected, knock-down of KIF20A expression exhibited significantly suppressed mobility compared with vector control cells (Fig 6C).

## Discussion

KIF20A is overexpressed in various tumor types, including pancreatic cancer, bladder cancer, gastric cancer, melanoma, hepatocellular carcinoma and breast cancer [23–29]. Moreover, significant evidence indicates that overexpression of KIF20A promotes cell proliferation in a variety of tumor types, indicating that KIF20A is involved in the progression of cancer [23,27,28,30,31]. To our knowledge, this is the first report to assess the expression and association of KIF20A with the clinicopathological features of NPC.

KIF20A is overexpressed and represents a potential immunotherapeutic target in pancreatic cancer, and silencing KIF20A using a small interfering RNA inhibited the proliferation, motility and invasion of pancreatic cancer cell lines [23]. Exertier et al. reported KIF20A protein was induced during vascular endothelial growth factor-A (VEGF-A)-stimulated angiogenesis in vivo[30]. Shi et al. identified KIF20A is a downstream target gene of Glioma-associated oncogene 2 (Gli2) via the Forkhead Box M1 (FoxM1)-MMB complex, and reported the Gli2-KIF20A axis plays an essential role in the growth and progression of hepatocellular carcinoma and KIF20A could serve as an important prognostic biomarker [27]. Yamashita et al. found KIF20A is a novel melanoma-associated antigen with potential as diagnostic and prognostic marker of melanoma [28]. Zou et al. demonstrated KIF20A has potential as both a prognostic factor and therapeutic target for endocrine therapy-resistant breast cancer [29]. KIF20A are believed to play a central role in mitosis during cell division through modulating microtubule dynamics. As we all know that the primary mechanism of action of the taxol/docetaxel is the disruption of microtubule dynamics through the stabilization of GDP-bound tubulin in the microtubule, thereby interrupting the process of cell division at mitosis. Furthermore, Khongkow and colleagues found paclitaxel targets the FoxM1-KIF20A axis to drive abnormal mitotic spindle formation and mitotic catastrophe, and reported deregulated expression of FoxM1 and KIF20A may confer resistance to paclitaxel in breast cancer; the authors suggested KIF20A may represent a prognostic biomarker and therapeutic target for overcoming taxane resistance in breast cancer [31].

Consistent with the research described above, the present study provides the first evidence that KIF20A is overexpressed and has clinical significance in NPC. KIF20A was both transcriptionally and translationally upregulated in NPC cell lines and clinical tumor specimens, in comparison with noncancerous nasopharyngeal epithelial cells and tissues. High KIF20A expression was significantly associated with gender, clinical stage, T category, N category, vital status and distant metastasis in the cohort of 105 patients with NPC, strongly indicating that this protein promotes the progression of NPC. Additionally, high KIF20A expression was associated with poorer 5-year OS and PFS in both the entire cohort of patients and the subgroups with T3–T4 disease, lymph node metastasis, clinical stage III–IV disease and CCRT subgroups. What’s more, we found that higher KIF20A expression was correlated with a significantly shorter OS and PFS in the advanced clinical stage III-IV NPC patients who treated with CCRT subgroup (*P* = 0.028 and *P* = 0.040, respectively; Fig 5C and 5D).The data demonstrated that the advanced clinical stage III-IV NPC patients who overexpress KIF20A still had a poor survival even after had treated with CCRT and KIF20A may have value as a biomarker to identify subgroups of patients that require aggressive treatment on the base of CCRT to improve the survival rate. Moreover, KIF20A was confirmed as an independent prognostic factor for OS and PFS after adjusting for other risk factors in multivariate analysis in the entire cohort and subgroup of patients with advanced clinical stage III-IV. Collectively, this data strongly suggests that KIF20A contributes to the development and progression of NPC.

Compared to other squamous cell carcinomas of the head and neck, NPC is characterized by a high tendency for metastatic dissemination [16]. In this study, KIF20A expression was significantly associated with distant metastasis (P = 0.001).

And knock-down of KIF20A expression strongly reduced migration and invasion in NPC cell lines. The ability of solid tumors to attract blood vessels (tumor angiogenesis) is one of the major rate-limiting steps of tumor progression [32]. Vascular endothelial growth factor A (VEGF-A) is an important VEGF family member that is essential for cell proliferation and migration [33–35]and is key regulator of embryonic and pathologic angiogenesis [36]. Critical associations between metastasis and overexpression of vascular endothelial growth factor (VEGF) exist in a variety of solid carcinomas, including NPC[37,38]. Expression analyses have indicated that several kinesin-encoding genes are upregulated in lymphoblasts and endothelial cells. Moreover, Exertier and colleagues reported induction of KIF20A *in vivo* in response to stimulation of angiogenesis with vascular endothelial growth factor-A (VEGF-A), and that mitosis-independent vascular outgrowth in aortic ring cultures was strongly impaired by inhibition of KIF20A protein[30]. On the basis of this evidence, we assume that high levels of KIF20A may promote migration and invasion in NPC via VEGF. However, further investigation is required to confirm this hypothesis.

The angiogenic switch represents a key event of tumor progression [32] and there has been much hope that anti-VEGF-A therapies may inhibit tumor growth. In practice, anti-VEGF strategies have limitations, associated with limited numbers of responders to therapy, the severe side effects of the VEGF-targeting antibody endostatin, and increasing concerns about the high costs of such treatments. However, as a major proangiogenic factor, it is of crucial importance to identify new druggable targets associated with the downstream functions of VEGF-A. The previously-described study by Exertier et al.[30] provided evidence that KIF20A lies downstream of VEGF signaling and mediates essential processes important for physiological and pathological vascular growth; therefore, KIF20A may constitute a potential novel target for anti-vascular tumor therapy. This knowledge may open up new therapeutic approaches, such as targeting kinesin inhibitors to the tumor endothelium and stroma to enhance the therapeutic efficacy of existing cancer treatments[39].

The first clinical evidence of the potential of kinesin inhibition as an anti-cancer strategy was reported in 2004[40]. A phase I clinical trial based in Japan combined a KIF20A-derived peptide with gemcitabine (GEM) in patients with advanced pancreatic cancer who had received prior chemotherapy and/or radiotherapy. No severe adverse effects (grade 3 or higher) related to the KIF20A-derived peptide occurred [41]. The KIF20A-derived peptide vaccine induced high numbers of IFN-γ-producing cells, even in patients treated with GEM, suggesting this combination therapy holds promise for advanced pancreatic cancer. Phase I/II clinical trials of cancer immunotherapy KIF20A-derived short peptides in lung cancer and cholangiocellular carcinoma are now currently underway. Encouragingly, Tomita et al [42] reported KIF20A-specific cytotoxic T lymphocytes (CTLs) were induced by stimulation with KIF20A-long peptides (LP) *in vitro* and *in vivo*. Significant KIF20A-specific T-helper type 1 (TH1) cell responses were detected in patients with malignant head-and-neck cancer receiving this immunotherapy (8/16, 50%). This evidence indicates induction of KIF20A-specific TH1 cells in response to KIF20A-LP vaccination may improve the clinical response to chemotherapy or other standard therapies[43–45]. Therefore, on the basis of the results of this evidence and the findings of this study, a clinical trial of KIF20A peptide–based immunotherapy should be considered for patients with NPC.

There are some limitations to our study. First, this was a retrospective study; second, our cohort size was not sufficient large for a robust interrogation of KIF20A as a prognostic marker. However, we should also notice that, based on the survival curve we have drawn, it may come to the same conclusion with the sample size increasing.

## Conclusions

In summary, this is the first study to report KIF20A is frequently overexpressed and has clinical significance in NPC, as high KIF20A expression was associated with significantly poorer OS and PFS. Moreover, assessing tumor expression of KIF20A may improve prognostication and help to identify patients who may benefit from more aggressive treatment. Our in vitro analyses demonstrated that migration and invasion of cancer cells were significantly reduced by silencing KIF20A.

## Abbreviations

CCRT: concurrent chemoradiotherapy
CI: confidence interval
CTLs: cytotoxic T lymphocytes
EBV: Epstein-Barr virus
FoxM1: Forkhead Box M1
GAPDH: glyceraldehyde-3-phosphate dehydrogenase
GEM: gemcitabine
Gli2: Glioma-associated oncogene 2
HNSCC: head and neck squamous cell carcinoma
HR: hazard ratio
IMRT: intensity-modulated radiotherapy
KIF20A: kinesin family member 20A
LP: long peptides
NPC: nasopharyngeal carcinoma
OS: overall survival
PFS: progression-free survival
UICC: Union for International Cancer Control
VEGF: vascular endothelial growth factor
